# The clinical characteristics, gene mutations and outcomes of myelodysplastic syndromes with diabetes mellitus

**DOI:** 10.1007/s00432-023-05591-4

**Published:** 2024-02-02

**Authors:** Fanhuan Xu, Jiacheng Jin, Juan Guo, Feng Xu, Jianan Chen, Qi Liu, Luxi Song, Zheng Zhang, Liyu Zhou, Jiying Su, Chao Xiao, Yumei Zhang, Meng Yan, Qi He, Dong Wu, Chunkang Chang, Xiao Li, Lingyun Wu

**Affiliations:** 1 Department of Hematology, Shanghai Sixth People’s Hospital Affiliated to Shanghai Jiao Tong University School of Medicine, Shanghai, 200233 China; 2 Department of Hematology, Shanghai Jiao Eighth People’s Hospital, Shanghai, 200233 China

**Keywords:** Myelodysplastic syndromes, Diabetes mellitus, Gene mutation, Prognosis, Infection

## Abstract

**Purpose:**

Diabetes mellitus (DM) is the second most common comorbidity in myelodysplastic syndromes (MDS). The purpose of the study was to investigate the clinical characteristics of MDS patients with DM.

**Methods:**

A retrospective analysis was performed on the clinical data of 890 MDS patients with or without DM. Clinical data, including genetic changes, overall survival (OS), leukemia-free survival (LFS) and infection, were analyzed.

**Results:**

Among 890 patients, 184 (20.7%) had DM. *TET2* and *SF3B1* mutations occurred more frequently in the DM group than those in the non-DM group (*p* = 0.0092 and *p* = 0.0004, respectively). Besides, DM was an independent risk factor for infection (HR 2.135 CI 1.451–3.110, *p* = 0.000) in MDS. Compared to non-DM patients, MDS patients with DM had poor OS and LFS (*p* = 0.0002 and *p* = 0.0017, respectively), especially in the lower-risk group. While in multivariate analysis, DM did not retain its prognostic significance and the prognostic significance of infection was maintained (HR 2.488 CI 1.749–3.538, *p* = 0.000).

**Conclusions:**

MDS patients with DM have an inferior prognosis which may due to higher infection incidence, with *TET2* and *SF3B1* mutations being more frequent in those cases.

**Supplementary Information:**

The online version contains supplementary material available at 10.1007/s00432-023-05591-4.

## Introduction

Myelodysplastic syndromes (MDS) are a group of clonal and heterogeneous diseases originating from hematopoietic stem cells, characterized by ineffective and dysplastic hematopoiesis, that have an increased risk of acute myeloid leukemia (AML) transformation (Garcia-Manero et al. 2020). MDS is an important hematological malignancy in the elderly and has a high comorbidity rate. It was reported that approximately 70% of MDS patients have one or more comorbidities (Sakatoku et al. 2019). Cardiovascular diseases are the most prevalent comorbidities, with diabetes mellitus (DM) coming in second (Bammer et al. 2014).

DM includes type 1 diabetes (T1DM), type 2 diabetes (T2DM), other specific types of diabetes and gestational diabetes mellitus (GDM) (Harreiter and Roden 2019). The tight relationship between DM and cancer has been acknowledged. Age, physical inactivity, and obesity are risk factors for both DM and cancer (Shi and Hu 2014; Kyriakou et al. 2021). Obesity is the primary risk factor for DM, and it also contributes to the morbidity of MDS in the elderly (Hamoudeh et al. 2016). Studies have suggested that the relationship between obesity and MDS may be mediated by fetuin-A, adiponectin, and leptin (Schwabkey et al. 2021). Overweight individuals were found to have lower secretion of adiponectin, which can inhibit proliferation of bone marrow (BM) mononuclear cells by suppressing the expression of bcl-2. Leptin can stimulate the proliferation of leukemia cells in vitro. The pathogenesis of T2DM is mainly due to insulin resistance. Adiponectin and leptin can regulate the sensitivity of insulin to induce insulin resistance, which, in turn, leads to DM (Dalamaga et. al 2013).

Comorbidities affect the prognosis of MDS, especially in lower-risk patients, and comorbidities increase the mortality of patients directly. In higher-risk patients, comorbidities may influence prognosis by interfering with therapies for MDS (Della et al. 2011). However, comorbidities have not yet been formally incorporated into common prognostic scoring systems, such as the revised International Prognostic Scoring System (IPSS-R) and molecular IPSS (IPSS-M). The MDS-CI scoring model proposed by Della et al. (2011) indicated the relationship of prognosis between comorbidities and MDS, which includes cardiovascular diseases, liver diseases, lung diseases, kidney diseases, and solid tumors. DM had no significant effect on MDS patients by multivariate analysis and was not included in the model. However, there is still a lack of various studies to demonstrate the association between DM and the prognosis of MDS.

More than 50 gene mutations have been found in 80–90% of MDS patients, and abundant genes play a part in the pathogenesis of DM. *TET2*, as one of the commonly mutated genes in MDS, has been found to be connected with insulin resistance (Fuster et al. 2020), but the correlation between the gene mutation and DM in MDS patients has not yet been clearly indicated.

Cytopenia, immune dysfunction, and immune damage followed by therapies such as hypomethylating agents (HMAs) or chemotherapy result in increased infection in MDS (Zhai et al. 2022).Interestingly, there remain contradictions about how the coexistence of DM and MDS affects infection. The study of Toma et al. (2012) indicated that comorbidities, including DM, increase infection in MDS. According to Sullivan et al. (2013), DM is not an independent prognostic factor for infection in MDS. However, the number of DM patients in Sullivan was relatively small. Therefore, to explore the impact of DM on MDS, a large sample study was designed. Eight hundred ninety (890) MDS patients were enrolled, and retrospective research was conducted in the current study to analyze the clinical characteristics, including molecular genetic changes, infection, and prognosis, of MDS combined with DM. We found that MDS patients with DM have a higher frequency of *TET2* and *SF3B1* mutations and an inferior prognosis, which may be caused by the higher incidence of infection.

## Materials and methods

### Patients

Patients who were diagnosed with MDS at Shanghai Jiao Tong University School of Medicine Affiliated Sixth People's Hospital from February 2009 to November 2021 were involved in this study. Patient characteristics, including age, sex, blood counts, serum ferritin, serum erythropoietin, BM blast percentage, disease subtype (according to the World Health Organization (WHO) 2016 classification for MDS), karyotype, gene mutation, blood glucose and infection, were recorded. According to the IPSS-R, we categorized all MDS patients into five groups. Very low-, low- and part of intermediate-risk patients were defined as the lower-risk group, and another part of intermediate-risk, high- and very high-risk patients were defined as the higher-risk group.

Patients were classified into two groups: the DM group and the non-DM group. The DM group included patients who were diagnosed with DM prior to the onset of MDS or during the treatment of MDS. Others were classified into the non-DM group. The DM group was further subclassified into two groups according to blood glucose control: a good blood glucose control group and a poor blood glucose control group. During the period of treatment, patients whose glycosylated hemoglobin (HbA1c) ≦ 7.0 were defined as having good blood glucose control; otherwise, they were categorized into the poor blood glucose control group (Rosenstock et al. 2018).

Follow-up time started from the date of the diagnosis of MDS and ended on May 1, 2022. Overall survival (OS) was defined as the date of diagnosis to the date of death, end of follow-up, or loss to follow-up. Leukemia-free survival (LFS) was defined as the time from disease diagnosis to progression to leukemia or death.

### Targeted gene sequencing

Thirty-nine genes (*ASXL1, ANKRD11, BCOR, CALR, CBL, CEBPA, DNMT3A, DDX41, ETV6, EZH2, FLT3, GATA2, IDH1, IDH2, ITIH3, JAK2, KIF20B, KIT, KRAS, MPL, NF1, NPM1, NRAS, PHF6, PTPN11, PTPRD, ROBO1, ROBO2, RUNX1, SETBP1, SF3B1, SRSF2, STAG2, TET2, TP53, U2AF1, UPF3A, WT1* and *ZRSR*2) were examined for mutations by MiSeq sequencing (Illumina, San Diego, CA, USA) in cDNA from BM mononuclear cells of patients. Across the entire cohort, the average depth of the targeted sequencing analysis was 1000-fold (825–3521 reads). High-probability somatic changes included single-nucleotide variants and indels. Variants with variable allele frequencies (VAFs) > 10% were all extracted and annotated using ANNOVAR. Further attention given to the variants identified in > 5 positive reads among > 10 total reads and variants which were synonymous or ambiguous were discarded (Li et al. 2020a, b).

### Statistical analysis

All data were statistically analyzed by SPSS 26.0 and GraphPad Prism 9.0. Continuous variables are described as medians (ranges), and categorical variables are described as numbers (%). Normally distributed data were compared by *t* tests, and nonnormally distributed data were compared by Mann‒Whitney U tests. Qualitative data were compared by the Chi-square test or Fisher’s exact test. The Kaplan‒Meier method was used in univariate analysis to analyze survival data, and the log-rank test was carried out to estimate the difference of statistic. Multivariate analyses were performed on any parameters that were significant to the level of *p* < 0.1 in the univariate analysis. A Cox proportional hazards regression model was used to compare the discrepancy in survival between groups, and logistic regression analysis was carried out to evaluate infection. A *p* value of < 0.05 indicated that the difference was statistically significant.

## Results

### The prevalence of DM in MDS and the characteristics of the patients

A total of 890 patients were included in the study. The patients’ characteristics are shown in Table 1. The median age of the 890 MDS patients was 60 years. There were 533 (59.9%) males and 357 (40.1%) females among them. A total of 828 (93%) patients were available for the IPSS-R. Sixteen (1.8%) were in the very low-risk group, 178 (20.0%) in the low-risk group, 328 (36.9%) in the intermediate-risk group, 192 (21.6%) in the high-risk group and 114 (12.8%) in the very high-risk group. Sixty-two (7.0%) patients were not stratified because of a loss of cytogenetic information.

**Table 1:** Clinical characteristics of MDS patients with diabetes mellitus (DM) or non-DM

Parameter	All patients *n* = 890	DM *n* = 184	Non- DM *n* = 706	*p*-value
*Sex, n (%)*				
Male	533 (59.9)	115 (62.5)	418 (59.2)	0.416
Female	357 (40.1)	69 (37.5)	288 (40.8)	
Median age, year (range)	60 (18–91)	63 (23–85)	59 (18–91)	0.000
WBC,10^9/L mean (range)	2.7 (0.3–51.9)	2.7 (0.6–17.5)	2.7 (0.3–51.9)	0.597
ANC, 10^9/L mean (range)	1.1 (0–28.8)	1.1 (0–16)	1.1 (0–28.8)	0.525
HGB, g/L (range)	69 (20–171)	67 (20–152)	70 (27–171)	0.238
PLT, 10^9/L mean (range)	45 (0–988)	50 (0–805)	43 (0–988)	0.265
Serum ferritin, ng/ml mean (range)	760 (6.9–18,671)	811 (19.2–8101)	694 (6.9–18,671)	0.020
*Serum erythropoietin, n (%)*				
< 500 mIU/ml	314 (35.3)	77 (41.8)	237 (33.5)	0.079
> 500 mIU/ml	472 (53.0)	91 (49.5)	381 (54.0)	
Not available	104 (11.7)	16 (8.7)	88 (12.5)	
BM blasts percentage, % (range)	2.7 (0–29)	2.5 (0–22)	2.5 (0–29)	0.553
*WHO 2016 subtype, n (%)*				
MDS-SLD	53 (6.0)	5 (2.7)	48 (6.8)	0.011
MDS-MLD	379 (42.6)	84 (45.7)	295 (41.8)	
MDS-RS	80 (9.0)	25 (13.6)	55 (7.8)	
MDS-EB-1	175 (19.7)	29 (15.8)	146 (19.7)	
MDS-EB-2	170 (19.1)	38 (20.7)	132 (19.1)	
MDS-5q	5 (0.6)	0 (0.0)	5 (0.7)	
MDS-U	28 (3.5)	3 (1.6)	25 (3.5)	
*IPSS-R risk category, n (%)*				
Very low	16 (1.8)	1 (0.5)	15 (2.1)	0.298
Low	178 (20.0)	43 (23.4)	135 (19.1)	
Intermediate	328 (36.9)	64 (34.8)	264 (37.4)	
High	192 (21.6)	45 (24.5)	147 (20.8)	
Very high	114 (12.8)	20 (10.9)	94 (13.3)	
Not available	62 (7.0)	11 (6.0)	51 (7.2)	
*Cytogenetic group, n (%)*				
Very good	9 (1.0)	4 (2.2)	5 (0.7)	0.277
Good	573 (64.4)	112 (60.9)	461 (65.3)	
Intermediate	185 (20.8)	44 (23.9)	141 (20.0)	
Poor	43 (4.8)	10 (5.4)	33 (4.7)	
Very poor	24 (2.7)	4 (2.2)	20 (2.8)	
Not available	56 (6.3)	10 (5.4)	46 (6.5)	
Incidence of leukemia transformation, n (%)	123 (13.8)	27 (14.7)	96 (13.6)	0.706
Time of leukemic transformation, month (range)	15 (0.3–106.8)	17.8 (0.4–72)	12.6 (0.3–106.8)	0.313
*Infection, n (%)*				
Yes	449 (50.4)	121 (65.7)	328 (46.5)	0.000
No	441 (49.6)	63 (34.3)	378 (53.5)	
Median overall survival, months	50.2	30.0	77.0	0.000

*ANC*, absolute neutrophil count; *BM* bone marrow; *DM* diabetes mellitus; *HGB* homoglobin; *IPSS-R* revised international prognostic scoring system; *MDS-SLD* MDS with single lineage dysplasia; *MDS-MLD* MDS with multilineage dysplasia; *MDS-RS* MDS with ring sideroblasts; *MDS-EB-1* MDS with excess blasts-1; *MDS-EB-2* MDS with excess blasts-2; *MDS-5q*, MDS with isolated (5q); *MDS-U* MDS unclassifiable; *PLT* platelet; *WBC* White blood cell count; *WHO* World Health Organization

The DM group consisted of 184 (20.7%) patients with a median age of 63 years, 115 (62.5%) males and 69 (37.5%) females. Compared with the non-DM group, patients in the DM group were older (*p* = 0.000), while the sex composition ratio and IPSS-R in the two groups were not significantly different.

Blood counts were not significantly different between the two groups. A total of 786 patients (88.3%) were evaluated for the level of serum erythropoietin (EPO). A total of 77 (41.8%) patients had an EPO level < 500 mIU/ml in the DM group compared to 237 (33.5%) in the non-DM group (*p* = 0.079). Serum ferritin was available from 839 patients, and patients in the DM group had a significantly higher serum ferritin level than patients in the non-DM group (811 ng/ml vs. 694 ng/ml, *p* = 0.020).

### Influence of DM on disease subtypes

**Fig. 1:**
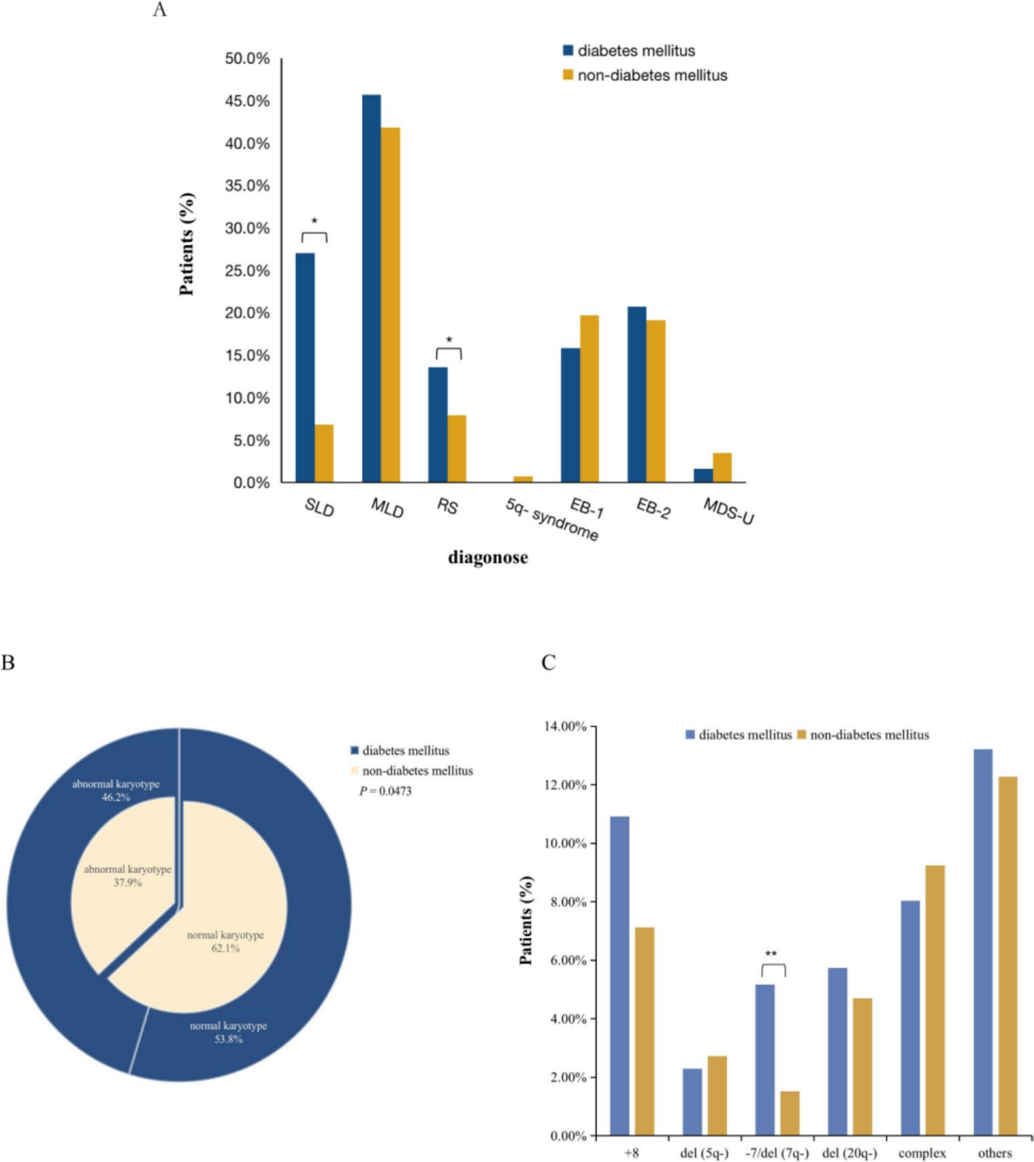
Disease subtypes and karyotypes of MDS patients with or without DM. **A** 2016 WHO classification in DM and non-DM group. **B** and **C** Karyotypes in DM and non-DM group (*p < 0.05,**p < 0.01)

According to the 2016 WHO classification for MDS, the most common subtype in the DM group was MDS with multilineage dysplasia (MDS-MLD) (42.6%), followed by MDS with excess blasts-1 (MDS-EB-1) (19.7%) and MDS with excess blasts-2 (MDS-EB-2) (19.1%). Remarkably, the DM and non-DM groups differed significantly in terms of disease subtypes (*p* = 0.011) (Fig. 1A). MDS with ring sideroblasts (MDS-RS) was more frequent in the DM group than in the non-DM group (13.6% vs. 7.8%, *p* = 0.0143). A lower incidence of MDS with single lineage dysplasia (MDS-SLD) was found in the DM group, compared to that in the non-DM group (2.7% vs. 6.8%, *p* = 0.0372). Other subtypes were not significantly different between the DM group and the non-DM group.

### Abnormal karyotypes were more common in the DM group

Abnormal karyotypes were more common in the DM group than in the non-DM group (46.2% vs. 37.9%, *p* = 0.0473) (Fig. 1B). Loss of chromosome 7 or deletion of the long arm of chromosome 7 (-7/del (7q)) (5.2% vs. 1.5%, *p* = 0.0038) occurred more frequently in the DM group than in the non-DM group (Fig. 1C). However, deletion of the long arm of chromosome 5 (del(5q)), deletion of the long arm of chromosome 20 (del (20q)), trisomy 8 (+ 8), complex and other karyotypes between the DM group and non-DM group were not significantly different. In addition, based on the IPSS-R karyotype classification, we classified patients into five cytogenetic subgroups and found that there was no significant difference between the two groups.

### Higher frequency of somatic genetic mutations in the DM group

**Fig. 2:**
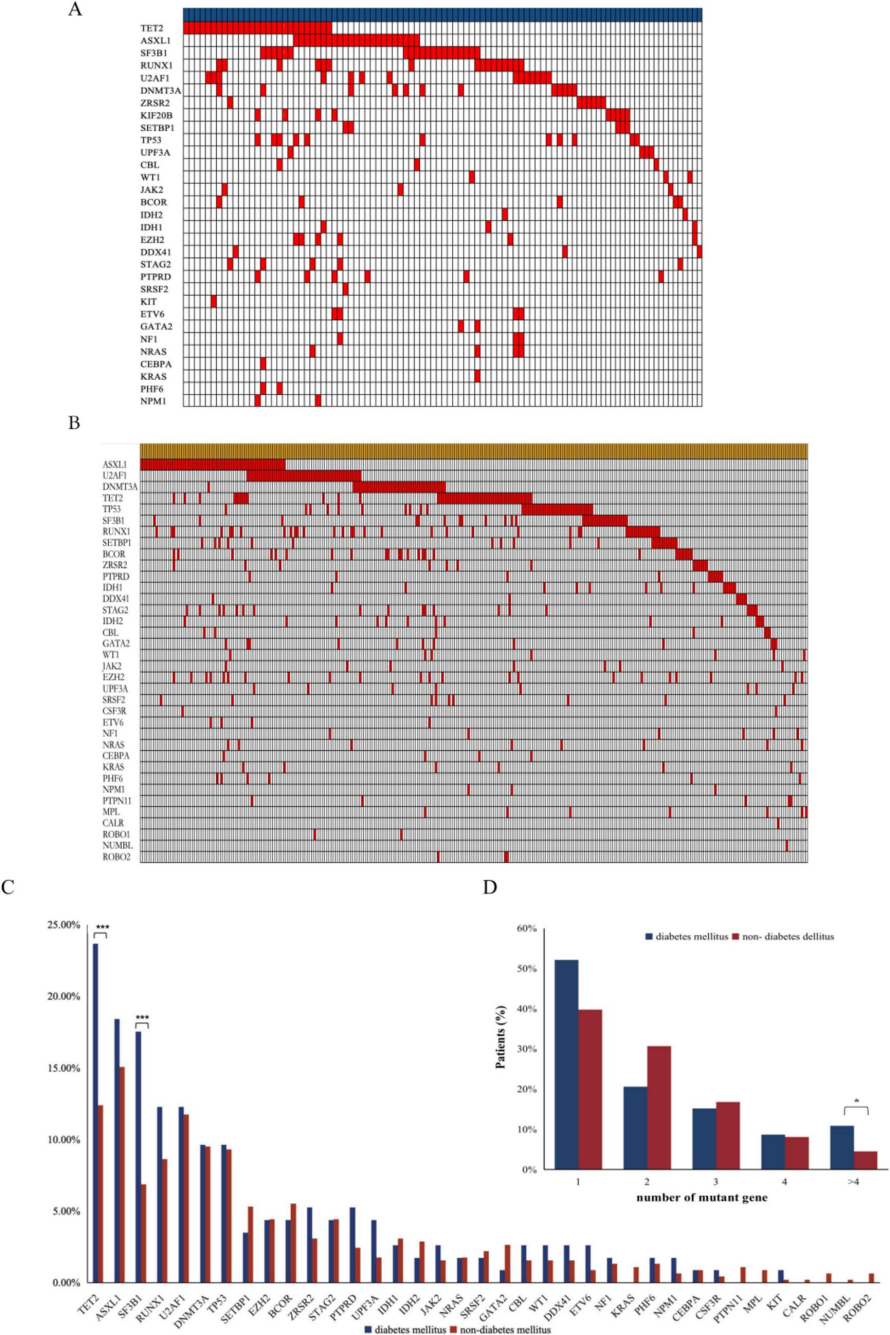
Mutation landscape of MDS. **A** Mutation landscape of DM group. **B** Mutation landscape of non-DM group. **C** Mutant gene type in DM and non-DM group. **D** Number of mutant gene of DM and non-DM group (*p < 0.05. ***p < 0.001)

Gene mutation data were available from 123 cases in the DM group and 445 cases in the non-DM group. A total of 98 of 123 (79.7%) patients in the DM group had at least one gene mutation, presenting a significantly higher frequency than that in the non-DM group (304 of 445, 68.3%, *p* = 0.0142). The number of mutated genes  between the two groups was also significantly different. Although the median number of mutant genes was similar, the percentage of patients with > 4 mutant genes was higher in the DM group than in the non-DM group (*p* = 0.0253) (Fig. 2D) The most frequently mutated genes in the DM group were *TET2*, *ASXL1*, *SF3B1*, *RUNX1* and *U2AF1*, while the most frequently mutated genes in the non-DM group were *ASXL1*, *U2AF1*, *DNMT3A*, *TET2*, and *TP53*. Noticeably, mutations in *TET2* (23.5% vs. 12.4%, *p* = 0.0092) and *SF3B1* (17.5% vs. 6.8%, *p* = 0.0004) were more common in the DM group than those in the non-DM group (Fig. 2C).

### Prognostic significance of DM in MDS

**Fig. 3:**
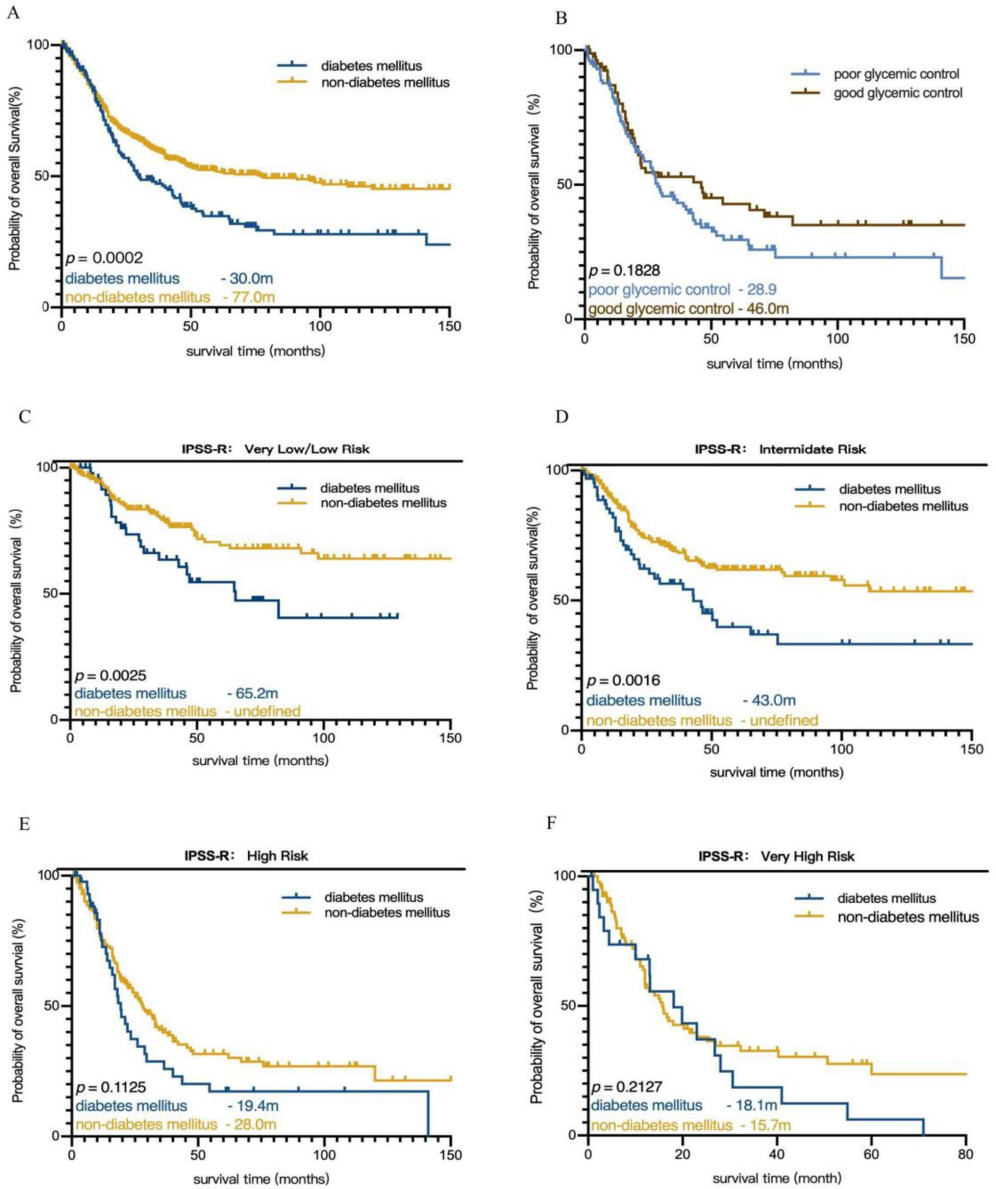
Kaplan–Meier cureves for OS and LFS of MDS patients with or without DM. **A** Kaplan–Meier cureves for OS of MDS. **B** Kaplan–Meier cureves for OS of MDS patients with DM. **C** Kaplan–Meier cureves for OS of MDS patients in very low- and low- risk group. **D** Kaplan–Meier cureves for OS of MDS patients in intermediate- risk group. **E** Kaplan–Meier cureves for OS of MDS patients in high- risk group. **F** Kaplan–Meier cureves for OS of MDS patients in very high- risk group

The median follow-up for the entire cohort was 50.2 (0–290) months, and there were 384 (43.1%) deaths. The median OS of patients in the DM group was 30 months, which was evidently shorter than that of the non-DM group (*p* = 0.0002) (Fig. 3A). After stratifying patients according to IPSS-R, we observed that DM was prognostic for shortened OS in patients with very-low/low-risk IPSS-R group [median OS, 65.2 (95% CI 26.5–104.0) months for patients with DM vs. not reached for those without DM] (*p* = 0.0025) (Fig. 3C) and in patients with intermediate-risk IPSS-R group [median OS, 43.0 (95% CI 23.4–62.7) months for patients with DM vs. not reached for those without DM] (*p* = 0.0016) (Fig. 3D). For patients with higher-risk IPSS-R group (high and very-high groups), there was no significant difference in OS between the DM group and the non-DM group (Fig. 3E and F). As for genetic mutations, six common mutant genes (*TP53*, *RUNX1*, *TET2*, *U2AF1*, *SF3B1*, and *ASXL1*) were included to the survival analysis, while the mutations of *RUNX1*, *U2AF1, SF3B1*, and *ASXL1* had no significant influence on OS. In the multivariate analysis, DM (HR 1.050 CI 0.765–1.442, *p* = 0.762) did not retained its prognostic significance in a Cox regression analysis model, while elderly age (HR 1.728 CI 1.255–2.379, *p* = 0.001), higher BM blasts percentage (HR 1.946 CI 1.438–2.632, *p* = 0.000), poor cytogengtic (HR 1.657 CI 1.256–2.187, *p* = 0.000), lower IPSS-R risk (HR 0.628 CI 0.405–0.974, *p* = 0.038), infection (HR 2.488 CI 1.749–3.538, *p* = 0.000), *TP53* mutation (HR 1.880 CI 1.222–2.893, *p* = 0.004), and *TET2* mutation (HR 1.565 CI 1.090–2.249, *p* = 0.005) affected on OS in all MDS patients independently (Table 2). Furthermore, we found that DM was an independent risk factor of prognosis without infection factor in the Cox regression analysis model (HR 1.514 CI 1.212–1.891, *p* = 0.000). However, when both DM and infection were put into the model, only infection remained its prognostic significance value for OS (HR 2.980 CI 2.374–3.740, *p* = 0.000). The median OS between the good blood glucose group and the poor blood glucose group was not significantly different (46.0 vs. 28.9 months, *p* = 0.1828) (Fig. 3B).

**Table 2:** Multivariate analysis for the overall survival of MDS

Parameter	*p*-value	HR (95%CI)
Sex	0.202	0.823 (0.610–1.110)
(male vs. female)		
Age	0.001	1.728 (1.255–2.379)
(> 60 vs. ≤ 60)		
ANC, 10^9/L	0.512	1.113 (0.807–1.535)
(< 0.8 vs. ≥ 0.8)		
*Cytogenetic group*		
Very poor	0.000	1.657 (1.256–2.187)
Poor		
Intermediate		
Good		
Very good		
BM blasts percentage,%	0.000	1.946 (1.438–2.632)
(> 10 vs.5–10 vs. > 2− < 5 vs. ≤ 2)		
Infection	0.000	2.488 (1.749–3.538)
*IPSS-R risk category*		
Very low	0.038	0.628 (0.405–0.974)
Low		
Intermediate		
High		
Very high		
Genetic mutations		
*TP53* (Mutation type vs. wild type)	0.004	1.880 (1.222–2.893)
*TET2* (Mutation type vs. wild type)	0.005	1.565 (1.090–2.249)
Diabetes mellitus	0.762	1.050 (0.765–1.442)
Serum erythropoietin, mIU/ml	0.318	1.194 (0.843–1.692)
(≤ 500 vs. > 500)		
HGB, g/L	0.057	0.741 (0.544–1.009)
(≥ 100 vs. 80− < 100 vs. < 80)		
PLT, 10^9/L	0.246	1.287 (0.840–1.971)
(< 50 vs. 50− < 100 vs. ≥ 100)		
Serum ferritin, ng/ml	0.318	1.194 (0.843–1.692)
(> 500 vs. ≤ 500)		

*ANC* Absolute neutrophil count; *BM* bone marrow; *HGB* homoglobin; *HR* hazard ratio; *IPSS-R* revised international prognostic scoring system; *PLT* platelet

**Fig. 4:**
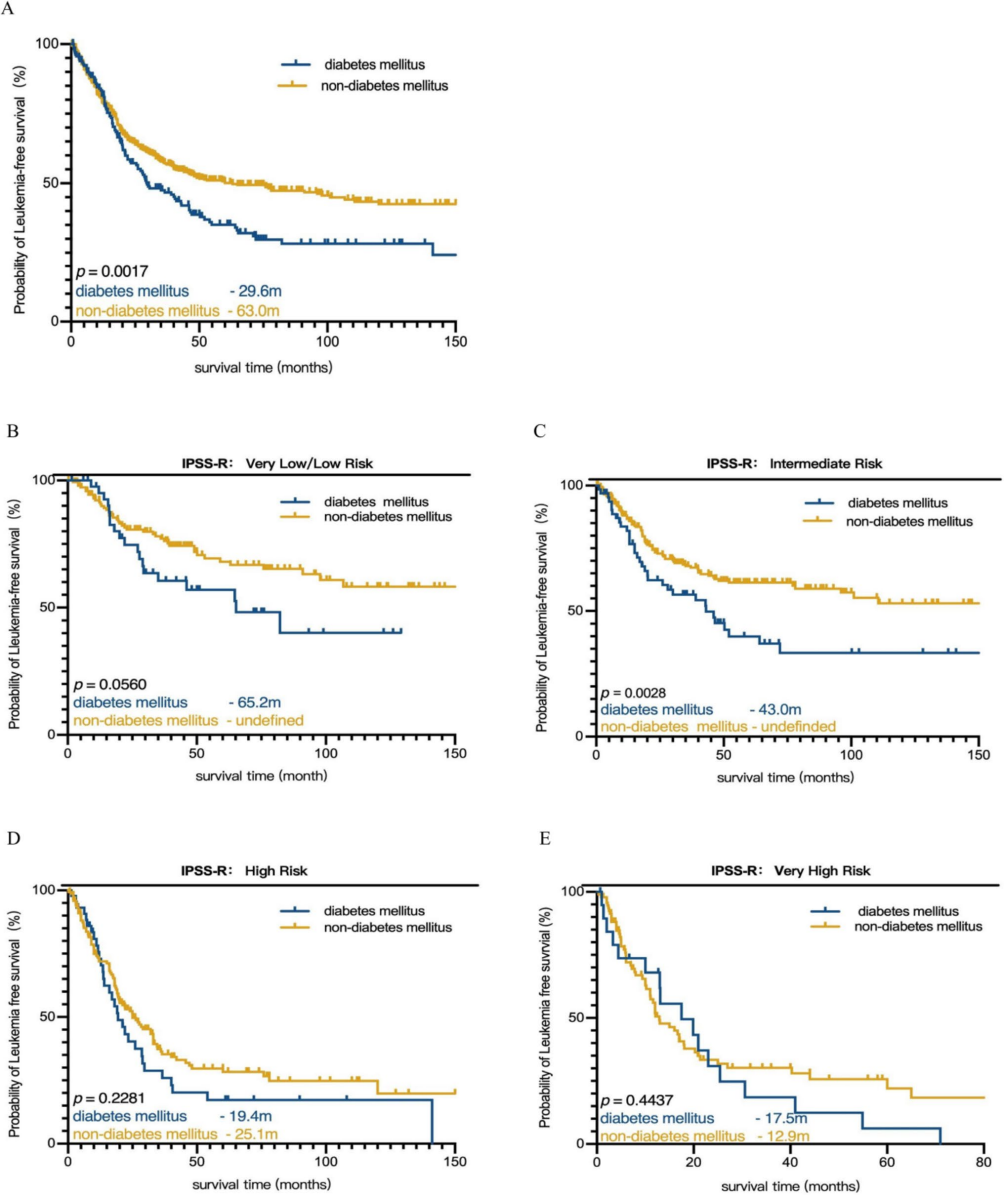
Kaplan–Meier cureves for LFS of MDS patients with or without DM. **A** Kaplan–Meier cureves for LFS of MDS. **B** Kaplan–Meier cureves for LFS of MDS patients in very low-/low- risk group. **C** Kaplan–Meier cureves for LFS of MDS patients in intermediate- risk group. **D** Kaplan–Meier cureves for LFS of MDS patients in high- risk group. **E** Kaplan–Meier cureves for LFS of MDS patients in very high- risk group

Moreover, DM was prognostic for LFS since patients with DM had a median LFS of 29.6 months, while the median LFS for patients without DM was 63 months (*p* = 0.0017) (Fig. 4A). The prognostic significance of DM for LFS was maintained in the intermediate-risk group [*p* = 0.0028 (Fig. 4C)]. Nevertheless, DM showed no significant effect on LFS in very low/low, high and very-high group (Fig. 4B, D and E). A total of 123 (13.8%) patients transformed to AML, including 27 (14.7%) patients who transformed to AML in the DM group and 96 (13.6%) patients in the non-DM group. There were no significant differences in either the AML transformation rate (14.7% vs. 13.6%, *p* = 0.706) or the median time of leukemia transformation (17.8 vs. 12.6 months,* p* = 0.313) between DM and non-DM MDS patients. Besides, all of the six common genetic mutations did not influence on the leukemia transformation significantly. While the *p*-value of *TET2* mutation < 0.1 (*p* = 0.091), so it was included in the multivariate analysis. In the Cox regression analysis, BM blasts (HR 2.337 CI 1.306–4.183, *p* = 0.004) and infection (HR 3.727 CI 1.910–7.270, *p* = 0.000) (Table 3) remained significant independent prognostic risk factors for AML transformation.

**Table 3:** Multivariate analysis for the leukemia transformation of MDS

Parameter	*p*-value	HR (95%CI)
Age, year	0.602	1.169 (0.641–2.098)
(> 60 vs. ≤ 60)		
ANC, 10^9/L	0.328	1.354 (0.737–2.488)
(< 0.8 vs. ≥ 0.8)		
HGB, g/L	0.705	1.113 (0.640–1.934)
(≥ 100 vs. 80− < 100 vs. < 80)		
PLT, 10^9/L	0.655	0.822 (0.348–1.942)
(< 50 vs. 50− < 100 vs. ≥ 100)		
Serum Erythropoietin, mIU/ml	0.929	1.030 (0.539–1.967)
(≤ 500 vs. > 500)		
Serum ferritin, ng/ml	0.144	0.626 (0.334–1.173)
(> 500 vs. ≤ 500)		
BM blasts percentage, %	0.004	2.337 (1.306–4.183)
(> 10 vs. 5–10 vs. > 2− < 5 vs. ≤ 2)		
*IPSS-R risk category*		
Very low	0.415	0.707 (0.307–1.629)
Low		
Intermediate		
High		
Very high		
*Cytogenetic group*		
Very poor	0.956	0.985 (0.585–1.659)
Poor		
Intermediate		
Good		
Very good		
*Genetic mutations*		
*TP53* (Mutation type vs. wild type)	0.109	2.016 (0.856–4.752)
Infection	0.000	3.727 (1.910–7.270)
Diabetes mellitus	0.774	0.909 (0.476–1.738)

*ANC* Absolute neutrophil count; *BM* bone marrow; *HGB* homoglobin; *HR* hazard ratio; *IPSS-R* revised international prognostic scoring system; *PLT* platelet; *WBC* White blood cell count

### DM was an independent risk predictor of infection in MDS

In the whole cohort, 72 (8.1%) patients suffered bacterial infection, 90 (10.1%) patients had fungal infection, 48 (5.4%) patients had both bacterial and fungal infection, and 239 (26.9%) patients got unknown pathogens infection (Supplementary Fig. 1). In the DM group, 61 (33.2%) patients experienced unknown pathogens infection, which was remarkably higher than that in non-DM patients (178 of 706, 25.2%, *p* = 0.030). Likewise, a significantly increased fungal infection rate of 17.4% (32 of 184) was observed in DM patients compared to 8.2% (58 of 706) in non-DM patients (*p* = 0.0002). Besides, both of the type and number of genetic mutations had no significant influence on infection (Supplementary Fig. 2). Ultimately, DM remained an independent risk factor for infection (HR 2.135 CI 1.451–3.110, *p* = 0.000) in MDS patients in multivariate analysis. In addition, age (HR 1.506 CI 1.120–2.086, *p* = 0.009), lower count of WBC (HR 1.451 CI 1.014–2.074, *p* = 0.041), higher serum ferritin (HR 1.613 CI 1.171–2.220, *p* = 0.003) and higher BM blasts percentage (HR 1.503 CI 1.287–1.755, *p* = 0.000) independently increased the risk of infection (Table 4).

**Table 4:** Univariate and multivariate analysis for infection of MDS

Parameter	Univariate	Multivariate	
	*p*-value	*p*-value	HR (95%CI)
Age, year	0.000	0.009	1.506 (1.120–2.086)
(> 60 vs. ≤ 60)			
WBC, 10^9/L	0.059	0.041	1.451 (1.014–2.074)
ANC, 10^9/L	0.025	0.721	0.983 (0.895–1.080)
(< 0.8 vs. ≥ 0.8)			
HGB, g/L	0.147		
PLT, 10^9/L	0.078	0.212	1.262 (0.875–1.820)
(< 50 vs. 50− < 100 vs. ≥ 100)			
Serum ferritin, ng/ml	0.001	0.003	1.613 (1.171–2.220)
(> 500 vs. ≤ 500)			
Erythropoietin, mIU/ml	0.219		
(≤ 500 vs. > 500)			
BM blasts percentage,%	0.000	0.000	1.503 (1.287–1.755)
(> 10 vs.5–10 vs. > 2− < 5 vs. ≤ 2)			
*IPSS-R risk category*			
Very low	0.000	0.634	1.038 (0.891–1.208)
Low			
Intermediate			
High			
Very high			
*Cytogenetic*			
Very poor	0.024	0.312	1.092 (0.921–1.296)
Poor			
Intermediate			
Good			
Very good			
Diabetes mellitus	0.000	0.000	2.135 (1.451–3.110)

*ANC* Absolute neutrophil count; *BM* bone marrow; *HGB* homoglobin; *HR* hazard ratio; *IPSS-R* revised international prognostic scoring system; *PLT* platelet; *WBC* White blood cell count

## Discussion

Comorbidities have a significant impact on the prognosis of hematological malignancies, including MDS. Although DM is the second most common comorbidity in MDS, there is ongoing debate concerning the prognostic significance of DM in MDS patients. Our study demonstrated that patients with MDS have a high incidence of DM, which has a negative impact on prognosis. In our cohort, 20.7% of MDS patients had DM, in line with previous literature (Goldberg et al. 2010), which is higher than the value of 12.8% of general adults in China (based on recorded data) (Li et al. 2020a, b). Fifty-three percent of the patients had DM after the attack of MDS, while 47% suffered from DM prior to MDS. In addition, elderly MDS patients have a higher risk of DM, which may be caused by loss of pancreatic function, less exercise, unhealthy sleep, etcetera (Wang et al. 2021). There are various causes for the high prevalence of DM in MDS patients. DNA methylation is one of the common changes in the epigenetics of MDS (Hosono 2019). Some studies have indicated that the dysregulation of DNA methylation, histone deacetylation and microRNAs may contribute to the incidence of DM in MDS (Bansal and Pinney 2017; Ahmed et al. 2020). DNA demethylation, which TET2 participates in, catalyzes the transformation of 5-methylcytosine (5mC) to 5-hydroxymethylcytosine (5hmC) (Gurnari et al. 2022). Our study also indicated that DM-MDS group has a significantly higher frequency of *TET2* mutations than that in the non-DM group. Loss-of-function mutations in *TET2* result in increased 5mC levels and lowered 5hmC levels. Previous studies have confirmed that the count of 5hmC is lower in DM patients, which has a negative correlation with HbA1C. In a mouse model, clonal hematopoiesis driven by somatic *TET2* mutation aggravates insulin resistance in mice (Fuster et al. 2020). Therefore, somatic *TET2* mutations may enhance the incidence of DM. DM patients who have *TET2* mutations may have abnormal regulation of the AMPK-TET2-5hmC pathway and would be more susceptible to cancer, including MDS (Wu et al. 2018; Villivalam et al. 2018). In addition, *TET2* plays a part in iron metabolism. Inokura et al. (2017) found that in zoopery, serum iron and serum ferritin increased in mice with *TET2* knockout. Moreover, there was a positive correlation between the level of serum ferritin and fasting glucose, glycosylated hemoglobin, and insulin resistance (Chen et al. 2017). Iron overload may emerge from ineffective hematopoiesis in erythroid cells, which may lead to increases in ferritin and then a reduction in the level of hepcidin. Moreover, iron overload could also result from blood transfusions, a routine therapy for MDS patients (Gattermann 2018). In our research, we also discovered a significant connection between serum ferritin levels and DM-MDS.

In our study, we found that abnormal karyotypes were more common in the DM group than that in the non-DM group, which supported the notion that DM patients might have a higher degree of chromosome instability (Cinkilic et al. 2009). Interestingly, the frequencies of -7/del(7q) were higher in the DM group than that in the non-DM group. It has been shown that genes such as the glucokinase (GCK) and also other genes located on chromosome 7 have correlations with DM (Rowe et al. 1995), but the relationship between -7/del(7q) and DM has not yet been well recognized.

Remarkably, the current study also showed that the incidence of gene mutations in DM-MDS patients was higher than that in the non-DM group. Previous research found that increased reactive oxygen species and Akt/tuberin signaling in DM accelerate DNA damage, which may increase the risk of gene mutations (Lee and Chan 2015). Gene mutations in MDS patients may have some correlation with the higher morbidity of DM. Except for *TET2* mutations, our study also indicated a significantly higher frequency of *SF3B1* mutations in the DM-MDS group than in the non-DM group. *SF3B1* is a gene that codes for splicing factor 3B subunit 1, which is most typically present in MDS-RS and mainly manifests as erythroid dysplasia and ineffective erythropoiesis (Malcovati et al. 2020). In comparison with non-*SF3B1* mutation, MDS patients with *SF3B1* mutation showed a significantly lower level of hemoglobin and relied more on blood transfusion. The *SF3B1* mutation leads to abnormal splicing of iron metabolism-related genes, which results in iron overload, ineffective erythropoiesis and transfusion dependence (Malcovati et al. 2020). Therefore, the iron overload as well as the higher serum ferritin from increased blood transfusion in patients with *SF3B1* mutation may increase the morbidity of DM. Accordance with the increased incidence of *SF3B1* mutation in DM-MDS patients, MDS-RS was also been observed to be more frequently in the DM group than that in non-DM group. Although a high glucose environment promotes gene mutation, the comutated gene number between the DM group and non-DM group was not significantly different.

In this study, we found that DM affected the OS of MDS patients, particularly in lower-risk patients, in contrast to the study of Wang et al. (2009), who proposed that DM had no impact on the OS of MDS patients. However, in multivariate analysis, DM did not retain its prognostic significance, same as the study of Della et al. (2011). While, we found that infection is an independent prognostic factor of OS in MDS. It was once thought that patients with lower-risk IPSS-R were more negatively impacted by comorbidities than those with higher-risk IPSS-R. This may be because for higher-risk patients, the severity of disease itself outweighed the impact of comorbidities, which accelerate the progression of MDS by raising the risk of nonleukemia death, such as cardiac diseases and infection (Sakatoku et al. 2019). A retrospective study in the United States showed that infection was a leading factor in lower-risk MDS patient fatalities. Reportedly, 38% of lower-risk MDS patients died from infection, compared with 15% die from AML transformation (Dayyani et al. 2010). We also discovered that DM is one of the independent risk predictors of infection and we supposed that higher risk of infection in DM-MDS patients may provided as a potential explanation for the poor prognostic of DM-MDS. A high glucose environment provides more nutrients for pathogenic bacteria, especially for gram-positive bacteria, and we can agree that bacterial infection is the most prevalent condition in MDS (Kim et al. 2020). As shown by Sullivan et al. (2013), hyperglycemia and DM were independent risk factors for pneumonia, urinary tract infection, and skin infection. Therefore, we are reminded to manage MDS patients with DM very carefully since the latter may increase the risk of infection, which could lead to nonleukemia death, although DM is not the only comorbidity that can lead to infectious mortality (Della et al. 2011).

Limitations in our research include the retrospective, single-center nature of the study and missing data related to cytogenetics, BM blasts, IPSS-R, all of which may have some influence on the outcomes. Besides, data of the numbers of infection occurrences, the site and severity of infection were not included in the current study. Moreover, the parameter selected to assess blood glucose control was not the best choice. Blood transfusion, hemorrhage, hemoglobinopathy and iron deficiency anemia may reduce HbA1c (Della et al. 2009), which has an impact on the statistics.

## Conclusions

In conclusion, the results of this study suggested that the morbidity of DM is higher in MDS patients, especially in elderly patients. MDS patients with DM showed a higher frequency of *TET2* and *SF3B1* mutations. In addition, DM has a poor influence on OS and LFS in MDS, particularly for lower-risk patients, as it may increase mortality by increasing the risk of infection which is an independent prognostic factor of OS, highlighting the necessity for blood glucose monitoring in MDS.

## Supplementary Information

Below is the link to the electronic supplementary material.

Supplementary file1 (DOCX 313 KB)

## Data Availability

The datasets generated during and/or analyzed during the current study are available from the corresponding author on reasonable request.
