# Supplementary material for: The clinical characteristics, gene mutations and outcomes of myelodysplastic syndromes with diabetes mellitus
Source: J Cancer Res Clin Oncol. 2024 Feb 2;150(2):71. doi: 10.1007/s00432-023-05591-4 (PMC10837231; doi:10.1007/s00432-023-05591-4)
Supplement: Supplementary file 1 — Supplementary file1 (DOCX 313 KB) [file 432_2023_5591_MOESM1_ESM.docx]

**The clinical characteristics, gene mutations and outcomes of myelodysplastic syndromes with diabetes mellitus**

**Journal:** Journal of Cancer Research and Clinical Oncology

**Authors:**

Fanhuan Xu^1^, Jiacheng Jin^1^, Juan Guo^1^, Feng Xu^1^, Jianan Chen^1^, Qi Liu^1^, Luxi Song^1^, Zheng Zhang^1^, Liyu Zhou^1,2^, Jiying Su^1^, Chao Xiao^1^, Yumei Zhang^2^, Meng Yan^2^, Qi He^1^, Dong Wu^1^, Chunkang Chang^1*^, Xiao Li^1*^, Lingyun Wu^1, 2*^

**Author affiliation:**

^1^Department of Hematology, Shanghai Sixth People's Hospital Affiliated to Shanghai Jiao Tong University School of Medicine

^2^Department of Hematology, Shanghai Jiao Eighth People's Hospital

^*^**Corresponding author:**

Lingyun Wu, MD, PhD, Department of Hematology, Shanghai Sixth People's Hospital Affiliated to Shanghai Jiao Tong University School of Medicine, Shanghai Jiao Eighth People's Hospital, Shanghai, 200233,China. Email: lincy2032@sjtu.edu.cn, Tel: +86-021-24058336, Fax: +86-021-64701361. Chunkang Chang, MD, PhD, Department of Hematology, Shanghai Sixth People's Hospital Affiliated to Shanghai Jiao Tong University School of Medicine, Shanghai, 200233,China. Xiao Li, MD, PhD, Department of Hematology, Shanghai Sixth People's Hospital Affiliated to Shanghai Jiao Tong University School of Medicine, Shanghai, 200233,China.

**Supplementary Fig. 1** Infection between the DM and non-DM group

**
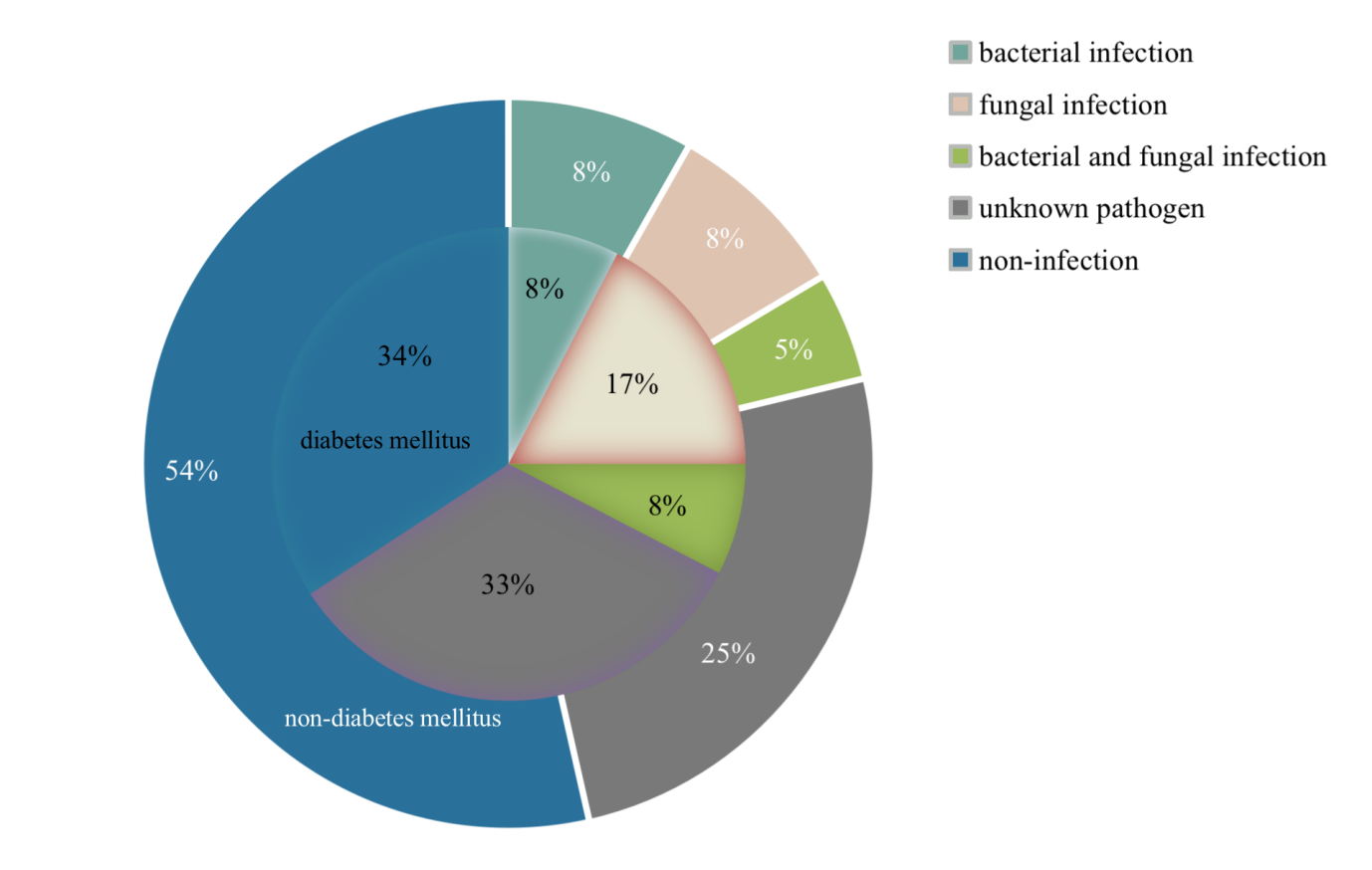
**


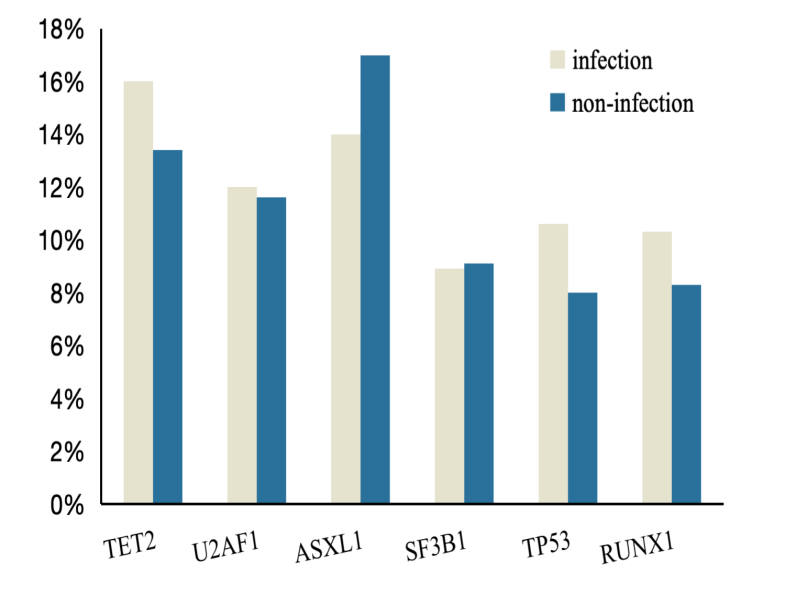

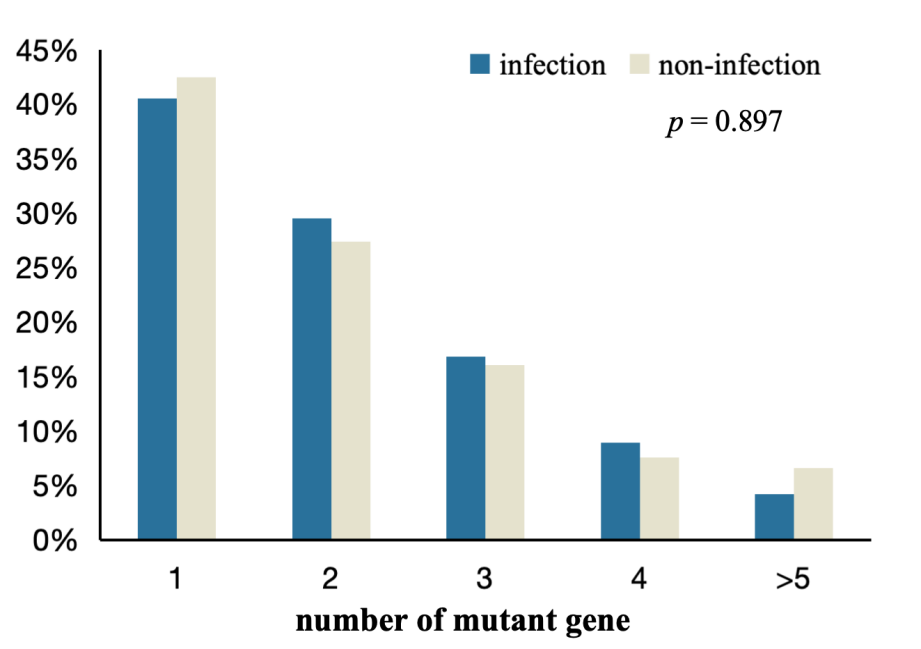


**Supplementary Fig. 2** Genetic mutation between the infection and non-infection group. A) Mutant

genetic type in infection and non-infection group. B) Number of mutant gene of infection and non-infection
